# Supplementary figures and images for: The commitment of barley microspores into embryogenesis correlates with miRNA‐directed regulation of members of the SPL, GRF and HD‐ZIPIII transcription factor families
Source: Plant Direct. 2020 Dec 8;4(12):e00289. doi: 10.1002/pld3.289 (PMC9671080; doi:10.1002/pld3.289)

MDS Plot: sRNA in microspores of barley cv.Gobernadora

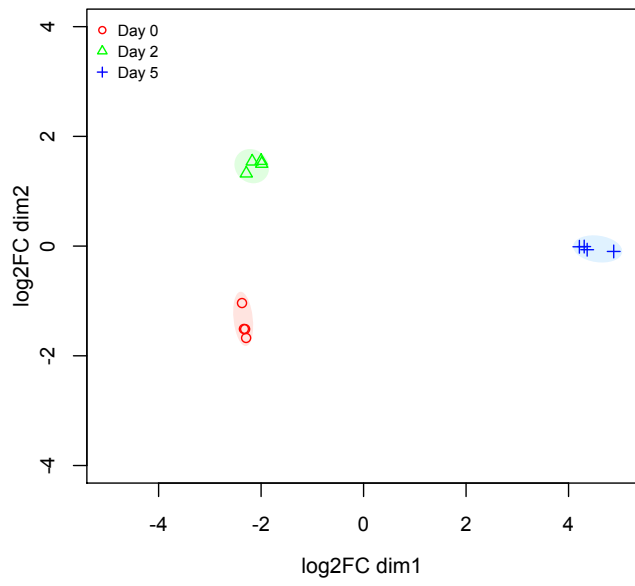

Supplement: Supplementary file 1 — Fig S1 [file PLD3-4-e00289-s005.pdf]
