# Supplementary material for: The commitment of barley microspores into embryogenesis correlates with miRNA‐directed regulation of members of the SPL, GRF and HD‐ZIPIII transcription factor families
Source: Plant Direct. 2020 Dec 8;4(12):e00289. doi: 10.1002/pld3.289 (PMC9671080; doi:10.1002/pld3.289)
Supplement: Supplementary file 2 — Table S1 [file PLD3-4-e00289-s004.xlsx]

Supplementary Table 1 The 5' and 3' adapters used to construct libraries.

	5' adapter	3' adapter
sRNA-seq	5'-GTTCAGAGTTCTACAGTCCGACGATC-3'	5'-AGATCGGAAGAGCACACGTCTGAACTCCAGTCAC-3'
PARE-seq	5'-GTTCAGAGTTCTACAGTCCGACGATC-3'	5'-TGGAATTCTCGGGTGCCAAGGAACTCCAGTCAC-3'
