# Supplementary material for: The commitment of barley microspores into embryogenesis correlates with miRNA‐directed regulation of members of the SPL, GRF and HD‐ZIPIII transcription factor families
Source: Plant Direct. 2020 Dec 8;4(12):e00289. doi: 10.1002/pld3.289 (PMC9671080; doi:10.1002/pld3.289)
Supplement: Supplementary file 3 — Table S2 [file PLD3-4-e00289-s003.xlsx]

Supplementary Table 2 Coordinates and abundance of the 202 PHAS loci detected in barley microspores (cv. Gobernadora) undergoing gametic embryogenesis.

PHAS loci ID	Chr	Start	End	Total abundance captured over 12 microspore libraries (rpm)
21PHAS_14322	3H	11468203	11468794	21.3
21PHAS_20868	3H	666495064	666495851	3.8
21PHAS_20987	3H	671295658	671296326	2.7
21PHAS_33492	5H	584990830	584991582	1.9
21PHAS_34003	5H	610464867	610465221	1.5
21PHAS_35755	6H	15137173	15137811	2.4
21PHAS_42227	7H	21074671	21075109	3.6
21PHAS_45781	7H	414260885	414264622	8.1
21PHAS_47766	7H	614055096	614055820	1.8
21PHAS_41814	7H	7355310	7355944	4.6
24PHAS_263	1H	13054359	13054962	25.6
24PHAS_268	1H	13068606	13070067	107.3
24PHAS_272	1H	13081265	13081853	39.6
24PHAS_46	1H	2112051	2112791	10.0
24PHAS_452	1H	21184712	21185479	20.8
24PHAS_472	1H	21635818	21636352	6.5
24PHAS_478	1H	21906855	21907639	5.6
24PHAS_480	1H	21912182	21912444	2.9
24PHAS_484	1H	22024537	22025274	12.0
24PHAS_492	1H	22164473	22165032	10.8
24PHAS_3397	1H	364128661	364132480	36.7
24PHAS_104	1H	4694003	4694589	9.4
24PHAS_4720	1H	487210157	487210328	58.9
24PHAS_4723	1H	487322711	487323442	253.0
24PHAS_112	1H	5141100	5142006	14.0
24PHAS_5591	1H	531451194	531452501	1.7
24PHAS_6022	1H	544721420	544721891	5.0
24PHAS_1120	1H	74270142	74270793	15.2
24PHAS_6878	2H	16876358	16877406	8.2
24PHAS_6891	2H	17054345	17055506	4.5
24PHAS_6895	2H	17089892	17090399	5.7
24PHAS_6901	2H	17158984	17159895	3.0
24PHAS_9080	2H	188226877	188228839	331.8
24PHAS_6992	2H	19237294	19238107	11.4
24PHAS_7004	2H	19326424	19327410	3.4
24PHAS_7009	2H	19367354	19368148	5.6
24PHAS_7013	2H	19463512	19464618	8.7
24PHAS_7025	2H	19689880	19690467	7.1
24PHAS_7152	2H	24564837	24565433	18.6
24PHAS_7162	2H	24899805	24900585	21.0
24PHAS_7265	2H	29567069	29568231	4.0
24PHAS_7474	2H	38376025	38378231	182.6

24PHAS_1169	2H	586819680	586822269	248.9
24PHAS_1239	2H	663464067	663466596	53.4
24PHAS_6621	2H	6708667	6709785	2.8
24PHAS_1256	2H	679346270	679347678	2.4
24PHAS_6627	2H	6991738	6992043	5.8
24PHAS_1318	2H	715835306	715835812	72.0
24PHAS_1328	2H	717666992	717667913	5.5
24PHAS_1346	2H	728781631	728782617	3.8
24PHAS_1348	2H	729376598	729377279	5.5
24PHAS_1348	2H	729400706	729401490	42.0
24PHAS_1348	2H	729497742	729498130	8.7
24PHAS_1348	2H	729558100	729558949	7.4
24PHAS_1368	2H	744773597	744774368	6.5
24PHAS_1380	2H	751202233	751203753	20.0
24PHAS_1385	2H	753195658	753196020	3.9
24PHAS_1385	2H	753200440	753201076	1.6
24PHAS_1389	2H	755104957	755106046	7.1
24PHAS_1398	2H	760732391	760733419	1.7
24PHAS_1401	2H	762094431	762095616	1.7
24PHAS_1402	2H	762116991	762118168	1.8
24PHAS_1409	2H	764844581	764845875	13.5
24PHAS_1410	2H	765730662	765732083	251.3
24PHAS_1411	2H	765919784	765920827	4.8
24PHAS_1435	3H	11740486	11741320	15.4
24PHAS_1438	3H	12085831	12086703	4.2
24PHAS_1438	3H	12120475	12121345	14.1
24PHAS_1438	3H	12123138	12123852	7.4
24PHAS_1439	3H	12294741	12295148	2.3
24PHAS_1439	3H	12296082	12297280	3.5
24PHAS_1447	3H	16256234	16257111	2.8
24PHAS_1611	3H	185045546	185047175	102.2
24PHAS_1453	3H	18905936	18908130	3.6
24PHAS_1469	3H	26975711	26976984	3.7
24PHAS_1712	3H	314356585	314358472	27.8
24PHAS_1420	3H	3661188	3662229	5.8
24PHAS_1874	3H	525824091	525824462	42.1
24PHAS_1517	3H	63340143	63340569	74.9
24PHAS_2071	3H	664150651	664151323	4.3
24PHAS_2072	3H	664327397	664327804	4.5
24PHAS_1520	3H	66434284	66436103	3.0
24PHAS_2072	3H	664359557	664360104	3.7
24PHAS_2091	3H	667876276	667877972	6.7
24PHAS_2121	3H	683875004	683877450	2.3
24PHAS_2121	3H	683899084	683899910	21.1
24PHAS_2122	3H	683902316	683903381	8.1
24PHAS_2123	3H	684159299	684160072	58.9
24PHAS_2123	3H	684171527	684173251	23.6

24PHAS_2124	3H	684233486	684234316	3.2
24PHAS_2124	3H	684242678	684243510	6.1
24PHAS_2124	3H	684372625	684373460	17.5
24PHAS_2125	3H	684381656	684382642	9.8
24PHAS_2125	3H	684389081	684390158	9.8
24PHAS_2157	3H	698405608	698406032	19.1
24PHAS_1425	3H	7693336	7695103	2.4
24PHAS_2161	4H	1232114	1233156	5.8
24PHAS_2188	4H	12435409	12438601	70.7
24PHAS_2351	4H	169628030	169631418	95.3
24PHAS_2232	4H	37361049	37362224	15.0
24PHAS_2232	4H	37379486	37380118	9.8
24PHAS_2233	4H	37681972	37682964	40.4
24PHAS_2233	4H	37695386	37696271	10.1
24PHAS_2238	4H	43171499	43172430	43.0
24PHAS_2238	4H	43181935	43182742	30.1
24PHAS_2239	4H	43493799	43494612	32.7
24PHAS_2770	4H	622935559	622936823	15.8
24PHAS_2791	4H	633812349	633814825	24.9
24PHAS_2946	5H	109616707	109620398	39.5
24PHAS_3181	5H	453066126	453072802	228.6
24PHAS_3229	5H	507349064	507349976	23.9
24PHAS_3277	5H	548843429	548845358	2.4
24PHAS_3287	5H	553897889	553898723	12.4
24PHAS_3288	5H	553971675	553972443	3.2
24PHAS_3297	5H	556833630	556834202	10.4
24PHAS_2827	5H	5587310	5588002	39.9
24PHAS_2827	5H	5588957	5589947	6.0
24PHAS_3345	5H	582637654	582639849	7.3
24PHAS_3429	5H	623928857	623930166	2.9
24PHAS_3436	5H	626025368	626027216	182.2
24PHAS_2831	5H	6319371	6320356	13.4
24PHAS_3452	5H	632530990	632531931	25.6
24PHAS_2831	5H	6341068	6342316	22.5
24PHAS_2832	5H	6352728	6353329	14.7
24PHAS_2832	5H	6372769	6373300	3.8
24PHAS_2833	5H	6499566	6500489	8.4
24PHAS_3494	5H	652041234	652043511	2.8
24PHAS_2833	5H	6551607	6553132	17.5
24PHAS_3510	5H	659336027	659337316	7.3
24PHAS_3514	5H	660944471	660945690	5.2
24PHAS_3516	5H	661060785	661063176	4.0
24PHAS_2936	5H	94270838	94271647	45.9
24PHAS_3560	6H	10954184	10954730	5.6
24PHAS_3560	6H	10956100	10956941	21.0
24PHAS_3561	6H	10960288	10960790	18.2
24PHAS_3561	6H	11071879	11072528	15.1

24PHAS_35615	6H	11073419	11074092	29.8
24PHAS_35618	6H	11088920	11089614	44.1
24PHAS_35622	6H	11123413	11124257	23.4
24PHAS_35628	6H	11301466	11302319	8.8
24PHAS_35806	6H	16645266	16646341	2.4
24PHAS_36128	6H	36173676	36174384	6.6
24PHAS_36266	6H	44042823	44047172	97.6
24PHAS_40746	6H	543603160	543603685	4.8
24PHAS_40748	6H	543700616	543701081	14.7
24PHAS_40749	6H	543734455	543735267	21.3
24PHAS_40750	6H	543737752	543738859	6.4
24PHAS_40751	6H	543856342	543857069	7.2
24PHAS_40752	6H	543858757	543859066	4.7
24PHAS_40957	6H	555942455	555943569	2.5
24PHAS_41280	6H	570939408	570942261	6.7
24PHAS_41285	6H	571024040	571025511	18.7
24PHAS_41286	6H	571027635	571028320	2.7
24PHAS_41289	6H	571041270	571041644	84.6
24PHAS_36607	6H	70476310	70477145	19.1
24PHAS_41963	7H	11341442	11342513	3.9
24PHAS_41964	7H	11380125	11381193	3.8
24PHAS_42015	7H	14297407	14297945	13.7
24PHAS_42017	7H	14419777	14419974	10.7
24PHAS_42019	7H	14435981	14436570	12.5
24PHAS_42021	7H	14447177	14448617	5.3
24PHAS_42023	7H	14484536	14485400	4.0
24PHAS_42030	7H	14735397	14736383	11.8
24PHAS_42041	7H	14949791	14950623	35.7
24PHAS_42052	7H	15502548	15502916	2.3
24PHAS_42055	7H	15527552	15528404	4.0
24PHAS_42057	7H	15543784	15544259	9.6
24PHAS_42059	7H	15574368	15574914	11.2
24PHAS_42063	7H	15617409	15617717	12.4
24PHAS_42075	7H	15667079	15667886	9.5
24PHAS_42078	7H	15673681	15674595	71.8
24PHAS_42092	7H	15934967	15935551	4.1
24PHAS_42099	7H	16188506	16188795	6.0
24PHAS_42100	7H	16188973	16189373	4.9
24PHAS_42470	7H	32268202	32269098	4.4
24PHAS_42617	7H	41001346	41002354	23.0
24PHAS_42619	7H	41005367	41006409	8.7
24PHAS_42653	7H	43887144	43888996	17.3
24PHAS_42711	7H	46466089	46467935	4.7
24PHAS_47112	7H	566467182	566467765	9.0
24PHAS_47139	7H	568548901	568549595	11.3
24PHAS_47146	7H	568689134	568690021	3.7
24PHAS_47187	7H	571782434	571782975	5.5

24PHAS_47188	7H	571792628	571793469	30.9
24PHAS_47889	7H	619383864	619384720	130.7
24PHAS_48004	7H	625274352	625274927	1.8
24PHAS_48144	7H	632308011	632308901	3.2
24PHAS_48157	7H	633013157	633014228	3.7
24PHAS_48305	7H	636391193	636392080	2.6
24PHAS_48306	7H	636408765	636409414	3.9
24PHAS_48312	7H	636582711	636584183	5.1
24PHAS_48337	7H	637600962	637601806	9.5
24PHAS_48343	7H	637666684	637667402	21.3
24PHAS_48345	7H	637729899	637730788	11.8
24PHAS_48551	7H	643000930	643002647	5.5
24PHAS_48650	7H	645403322	645404928	4.0
24PHAS_48780	7H	649918353	649919340	4.3
24PHAS_48870	7H	652224124	652224805	7.2
24PHAS_49727	Un	101568653	101572067	14.6
24PHAS_49094	Un	9394321	9394968	2.3
24PHAS_49097	Un	9401319	9402253	4.5
24PHAS_49099	Un	9418578	9419171	5.9
