# Supplementary material for: The commitment of barley microspores into embryogenesis correlates with miRNA‐directed regulation of members of the SPL, GRF and HD‐ZIPIII transcription factor families
Source: Plant Direct. 2020 Dec 8;4(12):e00289. doi: 10.1002/pld3.289 (PMC9671080; doi:10.1002/pld3.289)
Supplement: Supplementary file 4 — Table S3 [file PLD3-4-e00289-s002.xlsx]

Supplementary Table 3 Coordinates and abundance of the 68 annotated miRNAs in barley microspores (cv. Gobernadora) undergoing gametic embryogenesis.

miRNA	Chromosome	Start	End	Total abundance captured over 12 microspore libraries
miR1127	2H	156304849	156306165	1.4
miR1130.1	1H	517537524	517539284	10.0
miR1130.2	5H	558876253	558877962	0.7
miR1432	2H	166771786	166771953	0.9
miR156.1	2H	623451730	623451867	14.8
miR156.2	3H	59655592	59655682	0.9
miR156.3	3H	59655786	59655875	7.0
miR156.4	5H	499573181	499573663	5.1
miR156.5	5H	499573813	499574078	2.4
miR156.6	6H	362942108	362943254	68.4
miR159	3H	14332438	14332747	24.6
miR166.1	1H	353507033	353507137	92.1
miR166.2	5H	513807917	513808017	2.5
miR166.3	5H	593550048	593550127	84.7
miR166.4	6H	539379081	539379103	1.6
miR166.5	6H	539379309	539379352	29.5
miR166.6	7H	558749092	558749512	40.6
miR167.1	4H	590940939	590941052	1.3
miR167.2	4H	591009157	591009266	1.4
miR167.3	5H	595203284	595203376	1.9
miR167.4	6H	136921506	136921623	5.9
miR167.5	Un	58632427	58632921	103.9
miR167.6	Un	58635472	58635548	0.5
miR168	6H	34582524	34582666	27.4
miR171	4H	617878928	617879096	1.5
miR1878	5H	479890906	479890931	2.0
miR319	3H	140433531	140433705	3.0
miR393	2H	756463338	756463486	0.6
miR394	3H	649738003	649739841	0.6
miR396	6H	564855150	564855213	1.1
miR398	3H	637805239	637806060	1.7
miR408	7H	622016838	622016929	0.6
miR444	2H	583382959	583383500	2.3
miR5048	7H	550741190	550742460	28.9
miR5049.1	3H	95702904	95703500	1.0
miR5049.2	1H	534280976	534281606	1.3
miR5051	4H	588904352	588906553	7.0
miR5071.1	3H	667572215	667572472	6.8
miR5071.2	3H	667603611	667603714	1.2
miR5071.3	4H	387684886	387685014	1.7
miR5071.4	5H	289162901	289163053	0.6
miR5071.5	6H	572661234	572661255	1.9
miR5083.1	1H	554520362	554520768	1.2
miR5083.2	1H	554613391	554613666	0.6
miR5139.1	2H	457339091	457339814	0.6
miR5139.2	3H	267694291	267694389	2.7
miR5139.3	3H	546431702	546431746	0.9
miR5139.4	5H	325277882	325277997	1.5
miR5139.5	7H	566782938	566783769	1.0
miR5139.6	Un	126464383	126464540	6.0

miR5144	2H	23937585	23939427	1.3
miR8175.1	4H	285327304	285327355	0.6
miR8175.2	4H	611607026	611607134	2.9
miR8175.3	7H	164139781	164139848	1.3
miR827	2H	620316184	620316763	3.9
miR9660.1	6H	558257705	558257876	4.3
miR9660.2	6H	558398762	558398935	4.5
miR9662	6H	104007160	104007445	735.9
miR9674.1	3H	638444020	638444191	3.0
miR9674.2	6H	15557530	15557700	13.4
miR9773.1	3H	673282151	673282417	16.6
miR9773.2	7H	329970778	329971374	0.7
novel.1	1H	6577445	6577556	1.0
novel.2	4H	192456595	192456819	1.9
novel.3	5H	471378581	471378848	0.7
novel.4	7H	532917020	532917319	4.7
novel.5	7H	575649579	575649830	5.7
novel.6	7H	593828495	593828636	0.9
