# Supplementary material for: The commitment of barley microspores into embryogenesis correlates with miRNA‐directed regulation of members of the SPL, GRF and HD‐ZIPIII transcription factor families
Source: Plant Direct. 2020 Dec 8;4(12):e00289. doi: 10.1002/pld3.289 (PMC9671080; doi:10.1002/pld3.289)
Supplement: Supplementary file 5 — Table S4 [file PLD3-4-e00289-s006.xlsx]

Supplementary Table 4 Sequences of mature miRNA, miRNA* and their putative precursors as well as

	miRNA	Reads	miRNA*	Reads
Novel.1	AGCACUUUGAAUUUAGGUUGAACU	239	UAGCACUUUGAAUUUAGGUUGAAC	8
Novel.2	CUAGGACUUUGAAUUUGGGUUGAA	281	UAGGACUUUGAAUUUGGGUUGAAU	26
Novel.3	AUUUUAAUUGACGCUCAAACG	109	UUUGAGCGUCAAUUAAUACGA	9
Novel.4	AAGGCCUGAAGCUAAGCAACGUGC	1069	ACGUUGCUUAGCUUCAAGCCUUG	17
Novel.5	UCGAGGAGCUCUGUACAAAAAAG	623	UUUUUGUACGAAGCUCCUCGAAU	7
Novel.6	UUGCGUCAUUUAAUUCGGAUC	152	UCGGAAUUAAUUGACGCAGCC	60

s their abundance detected in microspores of barley cv. Gobernadora undergoing gametic embyrogenesis.

Precursor sequence and structure

```
GUACGAAUUUAGAGACAAAAUUCAACCUAAAUUCAAACAGCUAGUUAGGCUAACUAGCACUUUAAAUUCAAAGAGCUAGUUAGGC
((.(((((((((((.....((((((((((((((((..(((((((((.(((((((((.((((......)))).))))))))))).)
UCGAAUUUAAAGACAAAAUUCAACCUAAAUUUAAAGUGCUAGUUAGCUUAACUUGCUAACUAGGACUUUGAAUUUGGGUUGAAUU
)((((((((.(((.(((((((((((((((((((((((.(((((((((.......))))))))).))))))))))))))))))))))
ACUAUUACUCCCUCCGUCCCAUUUUAAUUGACGCUCAAACGGAUGUAUCUAGCACUGAAAUACGUCUAGAUACAUCCGUUUGAGC
).((((((((((((((..(...((((((((((((((((((((((((((((.((........))))))))))))))))))))))))
GAGGCGCAGUGCUCACCUGCACGUUGCUUAGCUUCAAGCCUUGAGGAAGUGGUGUAGACCGCACGGAGCUAUCACGGUUUUGGAC
.)).))))))))))((((((((((((((((((((((.(((((((((((((((((((((.(((((((((((...((((((....
GUUUGGUAGUUAUAUAACAUUCGAGGAGCUCUGUACAAAAAAGACAAAAUCACUGAAAAUUAGUUAAAAUGUAAGUCACUGUUUG
((((((.((((.((((((((((((((((.(((((((((((.((((((.((..((((((..(((((..((.....))..)))))
UAGUUAGUUACUCCCUCCGAUCGGAAUUAAUUGACGCAGCCUCUAUAGAACGUAAAAUGGACAUUGUAUAGAGGUUGCGUCAUUU
...)))).(((((((((((((.(((((((.(((((((((((((((((.((.((.......)).)).))))))))))))))))).))
```

UAACUAGCACUUUGAAUUUAGGUUGAACUUCUCUCUAAAUUCGAAUCUA
))))))))..)))))))))))))))))....))))))))))))).)))..
UUCUCUAUAAAUUCGCAU
)).))).))))))))...
GUCAAUUAAUACGAAACGGAGGGAGUAGUAAUUG
)))))))))...)..)))))))))))))))...(
AAAAACUACACAUAGCUCCGUGCAGUCUACACUAUUUCCUCAAGGCCUGAAGCUAAGCAACGUGCGGGUG
.))))))....))))))))))))).)))))))))))))))))))))))).)))))))))))))))))))))))
AGCAGAUUUUGUAAUUUUGCCUUUUUUGUACGAAGCUCCUCGAAUGUUAUUUGACCACCAAACUU
)...)))))))).)))))).)))))))))))).))))))))))))))))).)))).)))))))))
AAUUCGGAUCGGAGGGAGUACCACUACUCA
))))).))))))))))))))))))))))))
