# Supplementary material for: The commitment of barley microspores into embryogenesis correlates with miRNA‐directed regulation of members of the SPL, GRF and HD‐ZIPIII transcription factor families
Source: Plant Direct. 2020 Dec 8;4(12):e00289. doi: 10.1002/pld3.289 (PMC9671080; doi:10.1002/pld3.289)
Supplement: Supplementary file 6 — Table S5 [file PLD3-4-e00289-s001.xlsx]

Supplementary Table 5 Detail of miRNA targets validated by PARE tags in barley micr

miRNA	Target gene				Degra
		Cleavage Position	Fragment Abundance	Category	P-value
Microspore on day 0					
miR1127	HORVU2Hr1G086380	1429	16	2	0.0047
miR1127	HORVU4Hr1G042610	3267	7	2	0.0446
miR1127	HORVU7Hr1G056530	64	5	2	0.0022
miR1127	HORVU7Hr1G059130	957	5	2	0.0051
miR168	HORVU7Hr1G007000	4986	12	2	0.0161
miR396	HORVU6Hr1G084590	2275	8	2	0.0316
miR5049.1	HORVU7Hr1G085310	3579	24	2	0.0090
novel.3	HORVU3Hr1G070880	2332	30	2	0.0029
Microspore on day 2					
miR167.3	HORVU2Hr1G121110	2360	3	0	0.0004

miR396	HORVU7Hr1G008680	174	45	2	0.0101
miR1127	HORVU2Hr1G086380	1429	48	0	0.0015
miR1127	HORVU2Hr1G070360	1224	31	0	0.0060
miR1127	HORVU3Hr1G051000	2868	21	2	0.0099
miR1127	HORVU7Hr1G097520	3060	13	2	0.0169
miR1127	HORVU3Hr1G078090	1532	48	0	0.0019
miR1130.1	HORVU6Hr1G057140	314	6	0	0.0010
miR1130.1	HORVU7Hr1G122690	130	6	2	0.0014
miR1130.1	HORVU5Hr1G044640	2241	7	2	0.0026

miR1130.2	HORVU4Hr1G064990	1255	51	2	0.0366
miR5048	HORVU7Hr1G043150	576	29	0	0.0018
miR5048	HORVU7Hr1G065130	516	29	0	0.0019
miR5049.1	HORVU7Hr1G085310	3579	25	2	0.0066
miR9674.1	HORVU0Hr1G031920	1487	11	0	0.0039
miR9674.1	HORVU0Hr1G035310	653	11	2	0.0250
miR9674.1	HORVU1Hr1G010890	1602	11	0	0.0026
miR9674.1	HORVU1Hr1G010970	1545	11	1	0.0058
miR9674.1	HORVU1Hr1G010990	824	11	0	0.0114

miR9674.1	HORVU1Hr1G011020	824	11	1	0.0072
miR9674.1	HORVU1Hr1G011150	1558	11	0	0.0026
miR9674.1	HORVU1Hr1G011160	1593	11	0	0.0029
miR9674.1	HORVU1Hr1G011250	888	11	0	0.0088
miR9674.1	HORVU1Hr1G011300	1667	11	1	0.0048
novel.3	HORVU3Hr1G070880	2332	46	2	0.0031
Microspore on day 5					
miR156.1	HORVU3Hr1G094730	897	7	1	0.0037
miR156.1	HORVU0Hr1G020810	845	4	2	0.0010
miR166.1	HORVU1Hr1G041790	1063	9	2	0.0171

miR167.3	HORVU2Hr1G121110	2360	4	2	0.0012
miR396	HORVU2Hr1G101770	666	10	2	0.0326
miR396	HORVU0Hr1G016590	324	10	2	0.0233
miR396	HORVU0Hr1G016610	571	10	0	0.0057
miR396	HORVU0Hr1G026650	851	10	2	0.0203
miR396	HORVU6Hr1G068370	840	10	2	0.0160
miR396	HORVU7Hr1G008680	174	324	0	0.0058
miR396	HORVU2Hr1G094470	597	4	0	0.0078
miR5048	HORVU7Hr1G043150	576	6	2	0.0308

miR5048	HORVU7Hr1G065130	516	6	2	0.0325
miR9674.1	HORVU0Hr1G031920	1487	18	0	0.0039
miR9674.1	HORVU0Hr1G035310	653	18	0	0.0050
miR9674.1	HORVU1Hr1G010890	1602	18	0	0.0026
miR9674.1	HORVU1Hr1G010970	1545	18	0	0.0029
miR9674.1	HORVU1Hr1G010990	824	18	0	0.0114
miR9674.1	HORVU1Hr1G011020	824	18	0	0.0036
miR9674.1	HORVU1Hr1G011150	1558	18	0	0.0026
miR9674.1	HORVU1Hr1G011160	1593	18	0	0.0029

miR9674.1	HORVU1Hr1G011250	888	18	0	0.0088
miR9674.1	HORVU1Hr1G011300	1667	18	0	0.0024
miR1127	HORVU7Hr1G056530	64	6	2	0.0037
miR1127	HORVU3Hr1G051000	2606	6	2	0.0054
miR1127	HORVU7Hr1G059130	957	6	0	0.0058
miR1127	HORVU1Hr1G060530	3168	8	2	0.0031
miR1127	HORVU7Hr1G097520	3060	14	2	0.0215
miR1130.1	HORVU5Hr1G044640	2241	7	2	0.0017
miR1130.1	HORVU4Hr1G045760	1719	5	2	0.0240

novel.3	HORVU3Hr1G070880	2332	35	2	0.0031

spores (cv. Gobernadora) undergoing gametic embryogenesis.

dome

Duplex	Orthologous gene	Orthologous gene symbol	Target function
5' AACAAGTATTTCCGGACGGAG '3<br>|||||||||||| ||||||| 3' AAGATCCTGTTCATAAAGGGCTGCCTCCCTCA '5	AT5G48930	HCT	Cell wall biogenesis/degradation, Auxin homeostasis
5' AACAAGTATTTCC-GGACGGAG '3<br>|||||||||||| o||||||| 3' AGGAGGCTGTTCATAAAGGTTCTGCCTCCCTC '5	AT5G19390	ROPGAP7	Signal transduction, Cell wall biogenesis
5' AACAAGTATTTCCGGACGGAG '3<br>|||||||||||||||||||| 3' AGGATACTGTTCATAAAGGCCTGCCTCCCTCA '5	AT1G66950	ABCG39	Transport
5' AACAAGTATTTCCGGACGGAG '3<br>||||||||||||| | |||| 3' AGGATACTGTTCATAAAGGCGTACCTCCCTCA '5	AT5G03880	MER24.18	Cell redox homeostasis
5' TCGCTTGGTGCAGATCGGGAC '3<br>| ||||||||||| |||||| 3' GTAGACCA-CGAACCACGTCGAGCCCTTGACAA '5	AT1G48410	AGO1	RNA-mediated gene silencing, Transcription, Transcription regulation, Translation regulation
5' TTCCACAGCTTTCTTGAACTT '3<br>|||||||||||o||o|| ||| 3' TTCCGAAAGGTGTCGAAGGAGCTGGAAACATT '5	AT1G79690	NUDT3	Hydrolase
5' ATTATTTAGGTACAGAGGAAG '3<br>|||||||||||||||| || 3' TGATGTTC-ATAAATCCATGTCTCCCTCATGCT '5	AT4G13360		Hydrolase, mitochondrial
5' ATTTTAATTGACGCTCAAACG '3<br>|| ||||||| |||||||||| 3' GCTAGGTATAATTAACAGCGAGTTTGCCTACA '5	AT3G16360	AHP4	Cytokinin signaling pathway, Two-component regulatory system
5' TGAAGCTGCCAGCATGATCTA '3<br>o ||||||||||| ||||||| 3' TGCTTATG-TTCGACGGTCGGACTAGATAGCCC '5	Os04g0671900	ARF8	Auxin signaling pathway, Transcription, Transcription regulation

```
5'      TTCCACA-GCTTTCTTGAACTT
'3
        ||||||| ||||||||||||
3' AACCTAAGGTGTCCGAAAGAACTTGCCAACGC
'5
```

Os06g0116200 GRF5 Transcription, Transcription regulation

```
5'       AACAAGTATTTCCGGACGGAG
'3
          |||||||||||| |||||||
3' AAGATCCTGTTCATAAAGGGCTGCCTCCCTCA
'5
```

AT5G48930 HCT Cell wall biogenesis/degradation, Auxin homeostasis

```
5'       AACAAGTATTTCCGGACGGAG
'3
          ||||||||||| ||||||||
3' AAGATCCTGTTCATAAAGCCCTGCCTCCTTCA
'5
```

AT5G50600 HSD1 Lipid biosynthesis, Lipid metabolism, Steroid biosynthesis

```
5'       AACAAGTATTTCCGGACGGAG
'3
          ||||||||||||||||||||
3' AAGATCCTGTTCATAAAGGCCTGCCTCCCTCA
'5
```

AT5G20970 HSP20

```
5'       AACAAGTATTTCCGGACGGAG
'3
          |||||||||||| | |||||
3' AAGATACTGTTCATAAAGGACCGCCTCCCTCA
'5
```

AT4G28470 RPN1B Protein catabolic process

```
5'       AACAAGTATTTCCGGACGGAG
'3
          ||||||||||| ||||||||
3' AAGATCCTGTTCATAAAGCCCTGCCTCCCTCA
'5
```

AT3G58830 T20N10_180

```
5'       TCTTATGTTGTGGGACGGAG-G
'3
         ||||||o||o|||||||||| |
3' TTTGCGAGAATATAATACCCTGCCTCACTCAT
'5
```

AT2G23360 FPP7 Unknown

```
5'       TCTTATGTTGTGGGACGGAGG
'3
         ||||||o||o|||||||||||
3' TTTGCGAGAATATAATACCCTGCCTCCCTCAT
'5
```

AT3G09920 PIP5K9 Carbohydrate metabolic process, Cellular amino acid metabolic process

```
5'       TCTTATGTTGTGGGACGGAGG
'3
         ||||||o||o|||||| ||||
3' TTTGCGAGAATATAATACCCTGGCTCCTTCAT
'5
```

AT5G13750 ZIFL1 Transport

```
5'       CTCTGTAACTTAATATAAGAC
'3
          ||||||||||||o||  |||
3' ATGTCTAAGACATTGAATTGTACACTGTTGTA
'5
```

AT2G32840 — Negative regulation of transcription by RNA polymerase II

```
5'       TATATTTGCAGGTTTTAGGTCT
'3
         |||||| ||||oo|||||||||
3' GTTCTCATATAATCGTCTGAAATCCAGAGCCA
'5
```

AT5G15730 — CRLK2 — Protein phosphorylation

```
5'       TATATTTGCAGGTTTTAGGTCT
'3
         |||||| ||||oo|||||||||
3' GTTCTCATATAATCGTCTGAAATCCAGAGCCA
'5
```

AT5G15730 — CRLK2 — Protein phosphorylation

```
5'        ATTATTTAGGTACAGAGGAAG
'3
            |||||||||||||||| ||
3' TGATGTTC-ATAAATCCATGTCTCCCTCATGCT
'5
```

AT4G13360 — Hydrolase, mitochondrial

```
5'       TTAAATTTCTCCATAGCATCA
'3
         |||||||||||||| |||||
3' TAGACTAATTTAAAGAGGTACCGTAGCCAGTT
'5
```

Os04g0350000 — Rf1 — Restorer of fertility

```
5'       TTAAATTTCTCCATAGCATCA
'3
         |||||||||||||| |||||
3' TAGACTAATTTAAAGAGGTACCGTAGCCAGTT
'5
```

Os04g0350000 — Rf1 — Restorer of fertility

```
5'       TTAAATTTCTCCATAGCATCA
'3
         |||||||||||||| |||||
3' TAGACTAATTTAAAGAGGTACCGTAGCCAGTT
'5
```

Os04g0351333 — Rf1 — Restorer of fertility

```
5'       TTAAATTTCTCCATAGCATCA
'3
         |||||||||||||| |||||
3' TAGACTAATTTAAAGAGGTACCGTAGCCAGTT
'5
```

Os10g0497300 — Rf1 — Restorer of fertility

```
5'       TTAAATTTCTCCATAGCATCA
'3
         |||||||||||||| |||||
3' TAGACTAATTTAAAGAGGTACCGTAGCCAGTT
'5
```

Os10g0497300 — Rf1 — Restorer of fertility

```
5'       TTAAATTTCTCCATAGCATCA
'3
         |||||||||||||| |||||
3' TAGACTAATTTAAAGAGGTACCGTAGCCAGTT
'5
```

Os04g0350000 Rf1 Restorer of fertility

```
5'       TTAAATTTCTCCATAGCATCA
'3
         |||||||||||||| |||||
3' TAGACTAATTTAAAGAGGTACCGTAGCCAGTT
'5
```

Os04g0350000 Rf1 Restorer of fertility

```
5'       TTAAATTTCTCCATAGCATCA
'3
         |||||||||||||| |||||
3' TAGACTAATTTAAAGAGGTACCGTAGCCAGTT
'5
```

Os04g0351333 Rf1 Restorer of fertility

```
5'       TTAAATTTCTCCATAGCATCA
'3
         |||||||||||||| |||||
3' TAGACTAATTTAAAGAGGTACCGTAGCCAGTT
'5
```

Os04g0350000 Rf1 Restorer of fertility

```
5'       TTAAATTTCTCCATAGCATCA
'3
         |||||||||||||| |||||
3' TAGACTAATTTAAAGAGGTACCGTAGCCAGTT
'5
```

Os04g0351333 Rf1 Restorer of fertility

```
5'       ATTTTAATTGACGCTCAAACG
'3
         || ||||||| ||||||||||
3' GCTAGGTATAATTAACAGCGAGTTTGCCTACA
'5
```

AT3G16360 AHP4 Cytokinin signaling pathway, Two-component regulatory system

```
5'       TGACAGAAGAGAGTGAGCAC
'3
         ||||||||||||| ||||||
3' CAACAGACTGTCTTCTCTCTCTCGTGTCAACA
'5
```

Os01g0922600 SPL2 Transcription, Transcription regulation

```
5'       TGACAGAAGAGAGTGAGCAC
'3
         ||||||||||||| ||||||
3' GACTCAACTGTCTTCTCTCTCTCGTGTCGACC
'5
```

Os08g0509600 SPL14 Transcription, Transcription regulation

```
5'       TCGGACCAGGCTTCATTCCCC
'3
         ○||||||||||||||○||
3' TGCTTAGGCCTGGTCCGAAGTAGGGTCCGTAG
'5
```

AT5G60690 REV Differentiation, Transcription, Transcription regulation

<table>
<tr><td>5' TGAAGCTGCCAGCATGATCTA '3
o ||||||||||| |||||||
3' TGCTTATG-TTCGACGGTCGGACTAGATAGCCC '5</td><td>Os04g0671900</td><td>ARF8</td><td>Auxin signaling pathway, Transcription, Transcription regulation</td></tr>
<tr><td>5' TTCCACA-GCTTTCTTGAACTT '3
||||||| ||||||||||||
3' ACGCAAAGGTGTCCGAAAGAACTTGCCAACGC '5</td><td>Os04g0600900</td><td>GRF3</td><td>Transcription, Transcription regulation</td></tr>
<tr><td>5' TTCCACA-GCTTTCTTGAACTT '3
||||||| ||||||||||||
3' ACGCAAAGGTGTCCGAAAGAACTTGCCAACGC '5</td><td>Os02g0701300</td><td>GRF4</td><td>Transcription, Transcription regulation</td></tr>
<tr><td>5' TTCCACA-GCTTTCTTGAACTT '3
||||||| ||||||||||||
3' ACGCAAAGGTGTCCGAAAGAACTTGCCAACGC '5</td><td>Os02g0701300</td><td>GRF4</td><td>Transcription, Transcription regulation</td></tr>
<tr><td>5' TTCCACA-GCTTTCTTGAACTT '3
||||||| ||||||||||||
3' ACGCAAAGGTGTCCGAAAGAACTTGCCAACGC '5</td><td>Os02g0701300</td><td>GRF4</td><td>Transcription, Transcription regulation</td></tr>
<tr><td>5' TTCCACA-GCTTTCTTGAACTT '3
||||||| ||||||||||||
3' ACGCAAAGGTGTCCGAAAGAACTTGCCAACGC '5</td><td>Os02g0701300</td><td>GRF4</td><td>Transcription, Transcription regulation</td></tr>
<tr><td>5' TTCCACA-GCTTTCTTGAACTT '3
||||||| ||||||||||||
3' AACCTAAGGTGTCCGAAAGAACTTGCCAACGC '5</td><td>Os06g0116200</td><td>GRF5</td><td>Transcription, Transcription regulation</td></tr>
<tr><td>5' TTCCACA-GCTTTCTTGAACTT '3
||||||| ||||||||||||
3' CGCCGAAGGTGTCCGAAAGAACTTGCCGGCGC '5</td><td>Os04g0574500</td><td>GRF12</td><td>Transcription, Transcription regulation</td></tr>
<tr><td>5' TATATTTGCAGGTTTTAGGTCT '3
|||||| ||||oo|||||||||
3' GTTCTCATATAATCGTCTGAAATCCAGAGCCA '5</td><td>AT5G15730</td><td>CRLK2</td><td>Protein phosphorylation</td></tr>
</table>

```
5'      TATATTTGCAGGTTTTAGGTCT
'3
        |||||| ||||oo|||||||||
3' GTTCTCATATAATCGTCTGAAATCCAGAGCCA
'5
```

AT5G15730 CRLK2 Protein phosphorylation

```
5'      TTAAATTTCTCCATAGCATCA
'3
        |||||||||||||| |||||
3' TAGACTAATTTAAAGAGGTACCGTAGCCAGTT
'5
```

Os04g0350000 Rf1 Fertility restorer

```
5'      TTAAATTTCTCCATAGCATCA
'3
        |||||||||||||| |||||
3' TAGACTAATTTAAAGAGGTACCGTAGCCAGTT
'5
```

Os04g0350000 Rf1 Fertility restorer

```
5'      TTAAATTTCTCCATAGCATCA
'3
        |||||||||||||| |||||
3' TAGACTAATTTAAAGAGGTACCGTAGCCAGTT
'5
```

Os04g0351333 Rf1 Fertility restorer

```
5'      TTAAATTTCTCCATAGCATCA
'3
        |||||||||||||| |||||
3' TAGACTAATTTAAAGAGGTACCGTAGCCAGTT
'5
```

Os10g0497300 Rf1 Fertility restorer

```
5'      TTAAATTTCTCCATAGCATCA
'3
        |||||||||||||| |||||
3' TAGACTAATTTAAAGAGGTACCGTAGCCAGTT
'5
```

Os10g0497300 Rf1 Fertility restorer

```
5'      TTAAATTTCTCCATAGCATCA
'3
        |||||||||||||| |||||
3' TAGACTAATTTAAAGAGGTACCGTAGCCAGTT
'5
```

Os04g0350000 Rf1 Fertility restorer

```
5'      TTAAATTTCTCCATAGCATCA
'3
        |||||||||||||| |||||
3' TAGACTAATTTAAAGAGGTACCGTAGCCAGTT
'5
```

Os04g0350000 Rf1 Fertility restorer

```
5'      TTAAATTTCTCCATAGCATCA
'3
        |||||||||||||| |||||
3' TAGACTAATTTAAAGAGGTACCGTAGCCAGTT
'5
```

Os04g0351333 Rf1 Fertility restorer

```
5'       TTAAATTTCTCCATAGCATCA
'3
         |||||||||||||| |||||
3' TAGACTAATTTAAAGAGGTACCGTAGCCAGTT
'5
```

Os04g0350000 Rf1 Fertility restorer

```
5'       TTAAATTTCTCCATAGCATCA
'3
         |||||||||||||| |||||
3' TAGACTAATTTAAAGAGGTACCGTAGCCAGTT
'5
```

Os04g0351333 Rf1 Fertility restorer

```
5'       AACAAGTATTTCCGGACGGAG
'3
          ||||||||||||||||||||
3' AGGATACTGTTCATAAAGGCCTGCCTCCCTCA
'5
```

AT1G66950 ABCG39 Transport

```
5'       AACAAGTATTTCCGGACGGAG
'3
          ||||||||||||||| ||||
3' AGGATACTGTTCATAAAGGCCTACCTCCCTCA
'5
```

AT5G20970 HSP20

```
5'       AACAAGTATTTCCGGACGGAG
'3
          ||||||||||||| | ||||
3' AGGATACTGTTCATAAAGGCGTACCTCCCTCA
'5
```

AT5G03880 MER24.18 Cell redox homeostasis

```
5'       AACAAGTATTTCCGGACGGAG
'3
          ||||||||||||||||||||
3' AGGATGCTGTTCATAAAGGCCTGCCTCCCTCA
'5
```

AT5G18860 NSH3 Response to jasmonic acid

```
5'       AACAAGTATTTCCGGACGGAG
'3
          |||||||||||| | |||||
3' AAGATACTGTTCATAAAGGACCGCCTCCCTCA
'5
```

AT4G28470 RPN1B Protein catabolic process

```
5'       TCTTATGTTGTGGGACGGAGG
'3
         ||||||o||o|||||| ||||
3' TTTGCGAGAATATAATACCCTGGCTCCTTCAT
'5
```

AT5G13750 ZIFL1 Transport

```
5'       TCTTATGTTGTGGGACGGAGG
'3
         ||||||o||o|||o|||||||
3' TTAGCAAGAATATAATACCTTGCCTCCCTCAT
'5
```

AT1G18660 mRNA processing

5' ATTTTAATTGACGCTCAAACG '3 || ||||||| |||||||||| 3' GCTAGGTATAATTAACAGCGAGTTTGCCTACA '5	AT3G16360	AHP4	Cytokinin signaling pathway, Two-component regulatory system

Stage of microspore

Day 0

Day 0

Day 0

Day 0

Day 0

Day 0

Day 0

Day 0

Day 2

Day 2

Day 2

Day 2

Day 2

Day 2

Day 2

Day 2

Day 2

Day 2

Day 2

Day 2

Day 2

Day 2

Day 2

Day 2

Day 2

Day 2

Day 2

Day 2

Day 2

Day 2

Day 2

Day 2

Day 2

Day 5

Day 5

Day 5

Day 5

Day 5

Day 5

Day 5

Day 5

Day 5

Day 5

Day 5

Day 5

Day 5

Day 5

Day 5

Day 5

Day 5

Day 5

Day 5

Day 5

Day 5

Day 5

Day 5

Day 5

Day 5

Day 5

Day 5

Day 5

Day 5

Day 5

Day 5
